# Standardising Anaesthetic Induction in Code Red Trauma Patients: A Quality Improvement Initiative at a Major London Trauma Centre

**DOI:** 10.7759/cureus.101769

**Published:** 2026-01-18

**Authors:** Amelia Hogan, Cosmo F Scurr

**Affiliations:** 1 Department of Anaesthesiology, Mater Misericordiae University Hospital, Dublin, IRL; 2 Department of Anaesthesiology, Imperial College Healthcare NHS Trust, London, GBR

**Keywords:** anaesthetic induction, haemorrhagic shock, major trauma, quality improvement project, visual aid

## Abstract

Introduction

'Code red' trauma patients, defined as haemodynamically unstable with ongoing haemorrhage, are at high risk of cardiovascular collapse during anaesthetic induction. This presents a particular challenge for anaesthetic trainees unfamiliar with major trauma protocols. We aimed to develop and implement a standardised anaesthetic induction cognitive aid for 'code red' trauma patients and to describe its feasibility, uptake, and perceived educational value within a major trauma centre.

Methods

We conducted a retrospective service evaluation at a major trauma centre in London. Sixty-five patients undergoing theatre-based anaesthetic inductions between 2019 and 2023 were identified. Pre- and post-induction mean arterial pressures (MAP) were recorded from anaesthetic charts to assess peri-induction hypotension. We designed a one-page visual 'Code Red Quick Reference Guide' subsequently refined through two Plan-Do-Study-Act (PDSA) cycles, incorporating feedback from teaching sessions.

Results

Fifty-one (79%) received ketamine and fentanyl; 14 patients (21%) received propofol and fentanyl. Post-induction hypotension was common, with a median MAP reduction of 37% within 15 minutes of induction. One patient required intraoperative cardiac massage. Following checklist implementation, trainees reported improved confidence, and senior clinicians perceived greater consistency in induction planning. The checklist was incorporated into departmental teaching.

Conclusions

This quality improvement initiative demonstrated the feasibility of implementing a trauma-specific anaesthetic induction cognitive aid within a major trauma centre. While post-intervention clinical outcome data were not collected, early informal feedback supported the ongoing use of the tool as part of departmental trauma education. This project highlights the potential role of low-cost cognitive aids in supporting anaesthetic practice during high-acuity trauma care.

## Introduction

Anaesthetising 'code red' trauma patients involves rapid decision-making in individuals with limited physiological reserve. Trainees unfamiliar with major trauma may not be confident in choosing appropriate induction drugs or managing hypotension secondary to haemorrhagic shock. Inappropriate drug selection can exacerbate haemodynamic instability.

Trauma remains a leading cause of early mortality in people under 50, with a particularly high burden in well-resourced health systems [1,2]. Anaesthesiologists play a central and expanding role in trauma care, including the delivery of prehospital anaesthesia, vascular access strategies, and haemodynamic stabilisation during resuscitation [3]. These responsibilities become more complex and time-critical in the context of haemorrhagic shock, where rapid physiological deterioration places immense pressure on decision-making [4,5].

One of the most high-risk interventions in this group is the induction of anaesthesia [5]. Hypovolaemic trauma patients often rely on preserved sympathetic tone to maintain central perfusion [4]. These patients can present with signs of significant shock (e.g., tachycardia, peripheral vasoconstriction, high lactate, and altered mental status) [6]. Traditional induction drugs such as propofol and opioids are poorly tolerated and may precipitate cardiovascular collapse [5]. 

Establishing timely and adequate vascular access presents a significant challenge [6]. These patients often have poor peripheral perfusion, limiting cannulation success. While central venous access can be beneficial, it should not delay operative haemorrhage control [6]. Therefore, our local practice favours the rapid insertion of two large-bore peripheral lines where possible.

There are additional considerations for patients who present with concomitant traumatic brain injury (TBI) [7]. In this population, maintaining cerebral perfusion pressure is vital, as even transient hypotension is associated with poorer neurological outcomes [8].

The NHS England Major Trauma Audit: National Report 2022 highlights the role of standardised protocols, early consultant involvement, and checklists in improving outcomes [9]. Cognitive aids such as checklists have demonstrated effectiveness in complex medical environments, improving consistency and reducing cognitive load [10]. 

Local observation at our major trauma centre identified variation in induction drug choice, airway management, and preparation among anaesthetic trainees. This variation, combined with the physiological vulnerability of 'code red' trauma patients, highlighted the need for a simple and structured induction support tool.

We therefore undertook a quality improvement initiative to develop and implement a trauma-specific anaesthetic induction cognitive aid. The primary aim was to describe the development and implementation of this tool within a major trauma centre. Secondary aims were to explore its feasibility, integration into existing trauma workflows, and perceived value for anaesthetic trainees and senior clinicians. This project was not designed to assess patient-level clinical outcomes or comparative effectiveness of induction agents [11].

## Materials and methods

This was a single-centre, descriptive quality improvement initiative conducted at St. Mary's Hospital, Imperial College Healthcare NHS Trust, London. The project combined a retrospective service evaluation with the development and implementation of a trauma-specific anaesthetic induction cognitive aid for 'code red' trauma patients. The retrospective service evaluation component was undertaken to characterise baseline practice and inform the development of the quality improvement intervention. The primary focus was to characterise baseline variation in anaesthetic induction practice, identify areas of risk, and assess the feasibility and perceived utility of a standardised induction support tool. The project was not designed to compare induction agents, assess patient-level outcomes following implementation, or perform statistical hypothesis testing.

This project was registered locally as a service evaluation and approved by the St. Mary's Imperial College Healthcare NHS Trust Audit Committee (reference 1152). In accordance with institutional policy, formal research ethics committee or IRB approval was not required, as the project involved retrospective review of anonymised data without deviation from standard clinical care. 

We retrospectively identified 65 'code red' trauma patients anaesthetised in theatre at St. Mary's Hospital between January 2019 and December 2023. Data were extracted from electronic records. All adult trauma patients undergoing emergency theatre-based induction under a 'code red' alert were included. Exclusions included prehospital anaesthesia and non-operative resuscitation.

Metrics included patient demographics and timing metrics, induction drugs and doses, blood products administered, and MAP values recorded at three time points: immediately before induction, five and 15 minutes after induction, and the lowest MAP value observed within 15 minutes. MAP was selected as a primary physiological measure due to its relevance to end-organ perfusion and its established use in trauma research [12].

**Figure 1:**
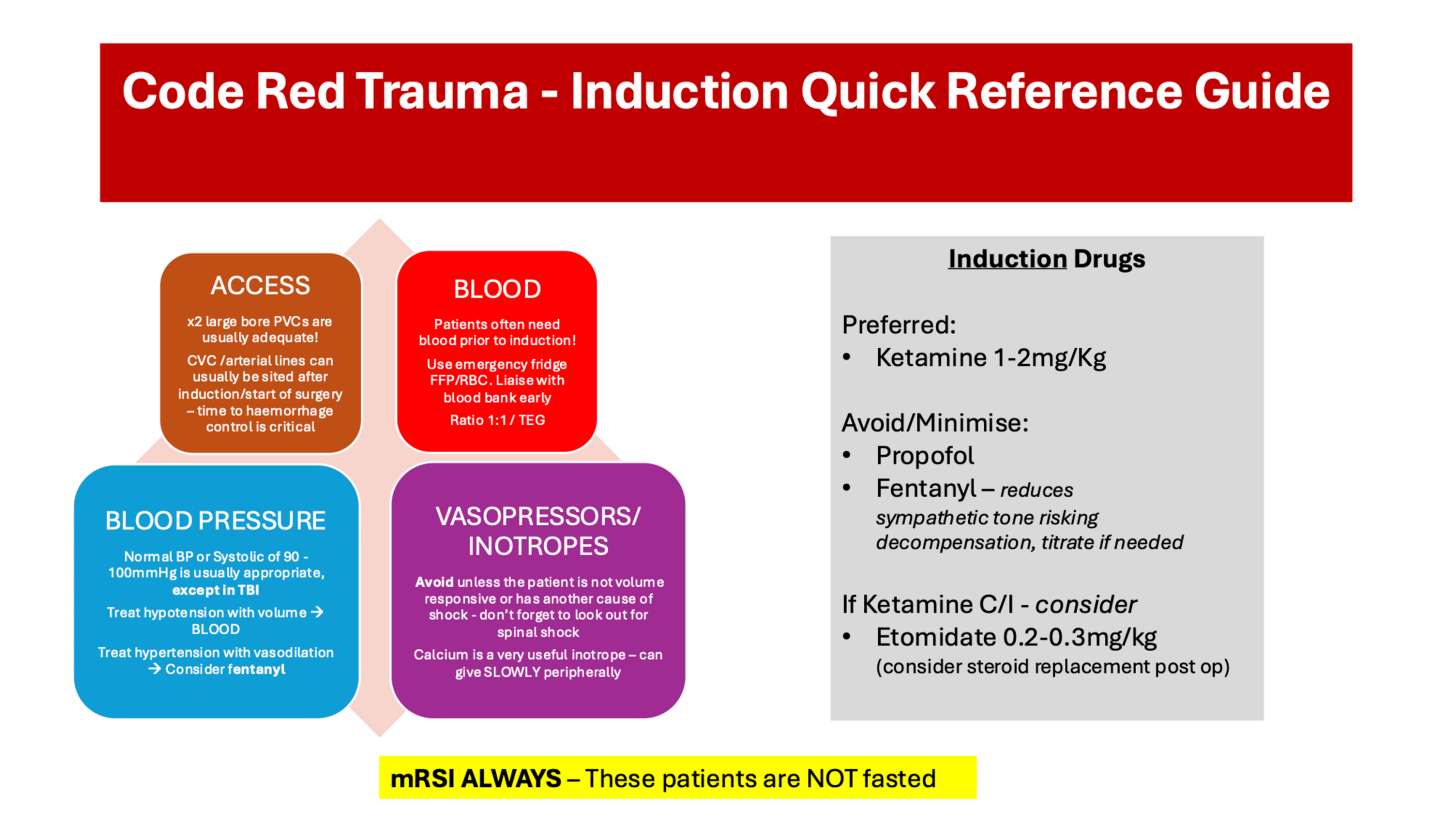
'Code red' induction cognitive aid Figure 1 presents a rapid-reference cognitive aid developed to support safe anaesthetic induction in haemodynamically unstable trauma patients. It emphasises prompt haemorrhage control and early resuscitation. Recommended induction agents include ketamine (1-2 mg/kg) or etomidate (0.2-0.3 mg/kg), with avoidance or cautious use of propofol and fentanyl due to their negative effects on sympathetic tone. mRSI is mandatory, as patients are assumed not to be fasted. Central venous access should not delay surgery unless deemed essential. MAP: mean arterial pressure; mRSI: modified rapid sequence induction; PVC: peripheral venous cannula; CVC: central venous catheter; BP: blood pressure; RBC: red blood cells; FFP: fresh frozen plasma; TEG: thromboelastography; TBI: traumatic brain injury Image Credit: Amelia Hogan

We designed a one-page visual aid poster aimed at anaesthetic trainees (Figure 1). The poster covered the following: preferred agents (e.g., ketamine), drugs to avoid or use cautiously (e.g., propofol, fentanyl), modified rapid sequence induction (mRSI) principles, aspiration risk, and early blood product administration. 

To support standardisation of practice, we incorporated principles of mRSI as described in contemporary airway guidelines. The Difficult Airway Society (DAS) 2015 guidelines emphasise that RSI should be adapted to patient physiology, particularly in haemodynamically unstable trauma patients [13]. Recommended modifications include the use of gentle mask ventilation where necessary to prevent hypoxaemia, adjustment of drug dosing to minimise cardiovascular compromise, and release of cricoid pressure if it obstructs ventilation or laryngoscopy. These recommendations informed the airway and drug selection strategies incorporated into our cognitive aid.

The poster was initially presented at departmental teaching and clinical governance meetings, where feedback highlighted the need for a clearer layout, simplified drug recommendations, and inclusion of trauma-specific considerations. Two Plan-Do-Study-Act (PDSA) cycles informed iterative refinements. In the first cycle, feedback from consultants and trainees led to improved visual clarity and streamlined content. In the second, supervised use during teaching sessions further refined the tool before final dissemination. Laminated copies were displayed in trauma theatres, and digital versions were incorporated into trainee induction materials. No formal pre- and post-intervention surveys or quantitative assessments of confidence were conducted. Feedback following implementation was collected informally during teaching sessions and clinical governance discussions and was used to guide the iterative refinement of the cognitive aid.

## Results

Results are presented descriptively and relate to baseline practice variation, peri-induction haemodynamic changes, and post-implementation staff feedback rather than to patient-level outcome comparisons.

The retrospective service evaluation found high variability in induction drug choices and significant post-induction hypotension. Fifty-one (79%) received ketamine and fentanyl; 14 (21%) received propofol and fentanyl (see Table 1). 

**Table 1:** Patient characteristics RCC: red cell concentrate; FFP: fresh frozen plasma

Characteristic	Result
Number of patients	65
Age, median (range), years	30 (16-67)
Pre-theatre RCC, median (range), units	2 (0-11)
Pre-theatre FFP, median (range), units	2 (0-12)
Induction drug combination, n (%)	
Ketamine + fentanyl	51 (79%)
Propofol + fentanyl	14 (21%)
Received blood products peri-induction, n (%)	44 (67%)

**Figure 2:**
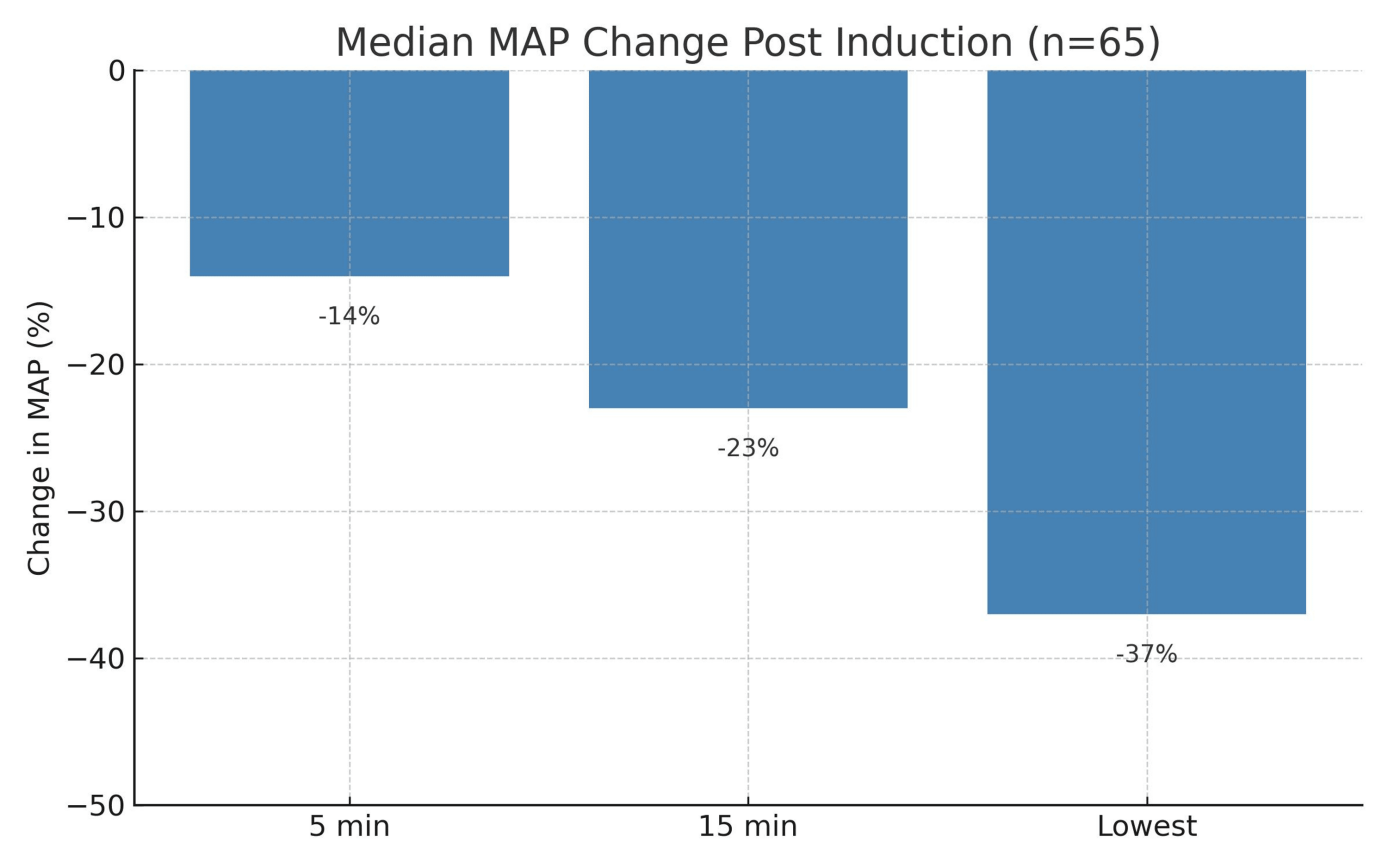
Median MAP change post-induction (n=65) Figure 2 displays the median percentage drop in MAP at key time points following anaesthetic induction in 65 'code red' trauma patients. Median values were −14% at five minutes, −23% at 15 minutes, and −37% for the lowest MAP recorded within the first 15 minutes. These findings highlight the haemodynamic instability of this patient cohort and the importance of anticipatory agent selection and early resuscitation. MAP: mean arterial pressure Image Credit: Amelia Hogan

MAP values demonstrated substantial early haemodynamic compromise. Median MAP changes were reductions by 14% at five minutes, 23% at 15 minutes, and 37% for the lowest recorded within 15 minutes of induction (see Figure 2). One patient deteriorated rapidly and required cardiac massage within 15 minutes, highlighting the severity of haemodynamic instability in this population. 

Post-project implementation, the visual aid was presented and discussed with trainees during local teaching sessions. Trainees reported improved confidence and preparedness, and senior staff felt it promoted consistent teaching and improved situational awareness.

Although quantitative outcome data were not collected following implementation, early feedback supported the continued use of the tool as part of departmental trauma education.

## Discussion

This project described variation in anaesthetic induction practice for 'code red' trauma patients, as demonstrated by differing induction drug combinations and peri-induction haemodynamic responses observed in the retrospective cohort. Retrospective audit data can help identify clinical risks and support targeted quality improvement. Simple interventions such as cognitive aid posters can enhance preparedness and encourage a more standardised approach. Departmental support and repeated engagement through teaching and governance meetings contributed to the successful adoption of the tool.

The haemodynamic findings in this study are consistent with existing trauma literature demonstrating that hypovolaemic patients are highly vulnerable to cardiovascular collapse following induction, particularly when traditional agents such as propofol or opioids are used [4-6]. The design and content of the cognitive aid are consistent with European trauma guidelines and prior studies that recommend ketamine or etomidate for induction in haemorrhagic shock due to their more favourable haemodynamic profiles [7]. The high proportion of ketamine use observed in this cohort is consistent with a broader shift in trauma anaesthesia practice away from agents with significant vasodilatory effects and towards drugs with more haemodynamically stable profiles, as described in contemporary trauma guidelines.

The introduction of the cognitive aid aligns with findings from Marshall showing that cognitive aids improve performance reliability during emergencies by reducing cognitive load and enhancing situational awareness [10]. Similar to other quality improvement interventions reported in the literature [14], structured visual tools may support user confidence and promote a shared cognitive framework during high-acuity clinical scenarios. In this respect, our findings are consistent with current evidence by demonstrating the perceived value of a trauma-specific induction aid within a UK major trauma centre.

This project has several important limitations. First, although retrospective physiological data were used to characterise peri-induction haemodynamic instability, no formal pre- and post-intervention patient-level outcome data were collected. As a result, the impact of the cognitive aid on clinical outcomes, drug selection, or haemodynamic parameters following implementation cannot be determined. Similarly, staff confidence, preparedness, and situational awareness were not measured using validated instruments, limiting interpretation to descriptive and subjective feedback.

Second, the initiative was conducted at a single major trauma centre with a relatively small sample size, which may limit generalisability to other institutions with different trauma workflows or staffing models. Feedback following implementation was informal and may be subject to reporting and confirmation bias. Checklist adherence during real-time clinical care was not prospectively monitored, making it difficult to directly correlate use of the tool with individual cases.

Finally, as a quality improvement initiative rather than a controlled comparative study, this project was not designed to evaluate differences between induction agents, assess the influence of variables such as Glasgow Coma Scale or haemorrhage severity, or determine statistical significance. Future work incorporating structured pre- and post-intervention data collection, multicentre collaboration, and prospective outcome assessment would be required to more rigorously evaluate the effectiveness of such interventions.

## Conclusions

This quality improvement initiative identified substantial variation in anaesthetic induction practice for 'code red' trauma patients and demonstrated the feasibility of implementing a simple, trauma-specific cognitive aid within a major trauma centre. While formal post-intervention outcome measurement was not undertaken, early informal feedback suggested improved preparedness and consistency in induction planning among trainees. This low-cost, easily implementable intervention may support safer anaesthetic practice in haemodynamically unstable trauma patients, with further prospective evaluation required to assess its clinical impact.
